# Surgical *versus* medical treatment for infective endocarditis in patients on dialysis: a systematic review and meta-analysis

**DOI:** 10.1080/0886022X.2022.2064756

**Published:** 2022-04-21

**Authors:** Sze-Wen Ting, Jia-Jin Chen, Tao-Han Lee, George Kuo

**Affiliations:** a Department of Dermatology, New Taipei City Municipal Tucheng Hospital, New Taipei City, ROC; b Department of Nephrology, Kidney Research Center, Linkou Chang Gung Memorial Hospital, Taoyuan, ROC; c Department of Nephrology, New Taipei City Municipal Tucheng Hospital, New Taipei City, ROC

**Keywords:** Dialysis, valvuloplasty, valve replacement surgery, infective endocarditis, mortality

## Abstract

Infective endocarditis (IE) is a serious infection and causes significant morbidity and mortality. However, the benefit of surgery for endocarditis besides antibiotic treatment in dialysis patients remains controversial. We performed a systematic review of studies published between 1960 and February 2022. Meta-analysis was conducted with a random-effects model to explore the in-hospital, 30, 60, 90, 180-d, and 1-year mortality rates in adult dialysis patients with IE. Sensitivity analysis, subgroup analysis, and meta-regression were performed to explore potential sources of heterogeneity. Confidence of evidence was evaluated by the GRADE system. Thirteen studies were included. The pooled odds ratio of in-hospital mortality was 0.62 (95% confidence interval [CI]: 0.30–1.28, *p* = .17), with moderate heterogeneity (*I*^2^ = 62%, *p* < .01). Three studies reported 30-d mortality, and the pooled odds ratio for surgery compared with medical treatment was even lower (0.36; 95% CI: 0.22–0.61, *p* < .01), with low heterogeneity (*I*^2^ = 0%, *p* = .86). With studies on fewer than 30 patients excluded, the sensitivity analysis revealed a low odds ratio of in-hospital mortality for surgery *versus* medical treatment (0.52; 95% CI: 0.27–0.99, *p* = .047), with moderate heterogeneity (*I*^2^ = 63%, *p* < .01). Subgroup analysis revealed no significant differences between any two comparator subgroups. Based on a very low strength of evidence, compared with medical treatment, surgical treatment for IE in patients on dialysis is not associated with lower in-hospital mortality. When studies on fewer than 30 patients were excluded, surgical treatment was associated with better survival.

## Introduction

Patients who have chronic kidney disease or are receiving dialysis therapy are at high risk of infection, related hospitalization, and mortality [1]. Infective endocarditis (IE) is a life-threatening infection that contributes to significant morbidity and death in patients undergoing dialysis. Compared with the general population, patients undergoing dialysis are more vulnerable to IE because of their impaired immunity, high prevalence of periodontal disorders, frequent vascular cannulation, and repetitive exposure to non-physiologic dialysates [2–4]. Among the general population, surgical indications for IE are clear with a moderate level of evidence. In patients on dialysis, however, the level of evidence is low, and recommendations are weak [5]. Patients undergoing dialysis have more comorbidities and higher perioperative complication risks than the general population. The Society of Thoracic Surgery score and EURO score both incorporate kidney function. Impaired kidney function or dialysis is a strong predictor of short-term mortality [6–8]. Because of the high perioperative risk, patients on dialysis who develop IE undergo surgical intervention less frequently than patients with IE and without kidney diseases [9]. Thus, evidence to support the benefit of surgery in such patients is lacking, and the decision regarding whether to perform surgery to treat IE continues to be made on a case-by-case basis.

This study systematically reviewed literature on the outcomes of patients with IE undergoing dialysis and compared the short-term and long-term mortality rates associated with surgical treatment in this population.

## Methods

### Literature search and data sources

We performed online literature searches of the PubMed, Embase, Medline, Cochrane, and ClinicalTrials.gov databases. In addition, we searched the OpenGrey, APA PsycNet, HMIC, and MedRxiv databases for gray literature. The most recent search was performed on 14 February 2022. The population, intervention, comparison, and outcome (PICO) framework of this study was as follows: The population was patients with acute IE on dialysis, the intervention was surgical treatment, the comparison was medical treatment alone, and the outcome was in-hospital mortality. The search strategy was designed to target published articles or conference abstracts containing ‘end stage renal disease’ and ‘infective endocarditis’ or related words. The protocol was registered in PROSPERO under the approval number CRD42021240700.

Two independent investigators (SW Ting and JJ Chen) performed a comprehensive search of the aforementioned sources for studies published through 14 February 2022. The details of the search strategies and the search results are summarized in Supplemental Tables 1–3.

### Study eligibility criteria

After eliminating duplicated studies, we used filters in EndNote and Excel software to screen titles and abstracts. Then, the studies were independently reviewed by two investigators (SW Ting and JJ Chen). Review articles, case reports, case series, and editorials were excluded, as well as articles with topics irrelevant to our PICO. We identified and excluded reports that used the same patient population used by another already included report and those published before 1960. To be eligible for this study, a study must (i) include an adult, chronic dialysis population with acute IE, (ii) and report in-hospital mortality separately for surgically and medically treated patients. Any disagreement between the two investigators was resolved through discussion with a third investigator (G Kuo) to reach a consensus. Studies were excluded if they had only a single treatment arm or if outcome information could not be retrieved from the full text.

### Data extraction and quality assessment

The data were extracted from each study independently by two investigators and included the publication year, study design, and whether patients with Duke criteria–based possible IE, prosthetic valve endocarditis, or recurrent IE or patients on peritoneal dialysis were excluded. The risk of bias of nonrandomized studies was assessed with the Newcastle–Ottawa Scale (NOS) in accordance with the suggestion of the Cochrane Group for Systematic Reviews (http://www.ohri.ca/programs/clinical_epidemiology/oxford.asp). We rated the risk of bias in each study as *high, moderate*, or *low* following a similar rationale in previous study [10]. The risk of bias was rated *low* if it got 3 or 4 stars in the selection domain, 1 or 2 stars in the comparability domain, and 2 or 3 stars in the outcome domain. *Moderate* risk of bias was determined if 2 stars in selection domain, 1 or 2 stars in comparability domain, and 2 or 3 stars in outcome domain. Eventually, if there was only 0–1 star in the selection domain, or 0 star in comparability domain or 0–1 star in the outcome domain, the risk of bias would be rated as high. Disagreements between the two initial investigators (SW Ting and JJ Chen) were resolved through consensus with the third author (G Kuo). The quality of evidence for the overall meta-analysis was assessed based on the guidelines of the Grading of Recommendations Assessment, Development and Evaluation (GRADE) Working Group methodology [11]. We presented the result of the quality of evidence assessment as a supplemental table by online GRADE Profiler [12].

### Outcome measures

The primary outcome of interest was in-hospital mortality for IE treated with surgery *versus* that treated with antibiotics alone. The secondary outcomes were 30, 60, 90, 180-d, and 1-year mortality after the index IE episode.

### Statistical analysis

Meta-analysis of the mortality rate was performed with a random-effects model because the criteria of patient selection differed among studies. The odds ratios (ORs) for mortality at the various time-points were analyzed with the *metabin* function of the meta package in R [13]. Heterogeneity was examined by using the *I*^2^ index, with *I*^2^ of <25%, 25–50%, and >50% indicating mild, moderate, and high heterogeneity, respectively. Sensitivity analyses were performed to assess the robustness of the results. Each sensitivity analysis was performed with studies enrolling fewer than 30 participants or studies presenting a high risk of bias excluded. Meta-regression was performed to explore the potential correlations between the study outcomes and patient numbers or study year. To explore possible sources of heterogeneity, subgroup analyses were performed to compare the treatment effects across the following variables: (i) exclusion of patients with Duke criteria–based possible IE, (ii) exclusion of patients on peritoneal dialysis, (iii) exclusion of patients with prior IE, (iv) exclusion of patients with prosthetic valve IE, and (v) study design (prospective *vs.* retrospective). Publication bias was assessed with a funnel plot and Egger’s test, with a *p* value of <.1 for Egger’s test indicating potential publication bias. Statistical analyses were performed with R version 4.1.0 (The R Foundation, Vienna, Austria) [14].

## Results

### Literature search

**Figure 1.**
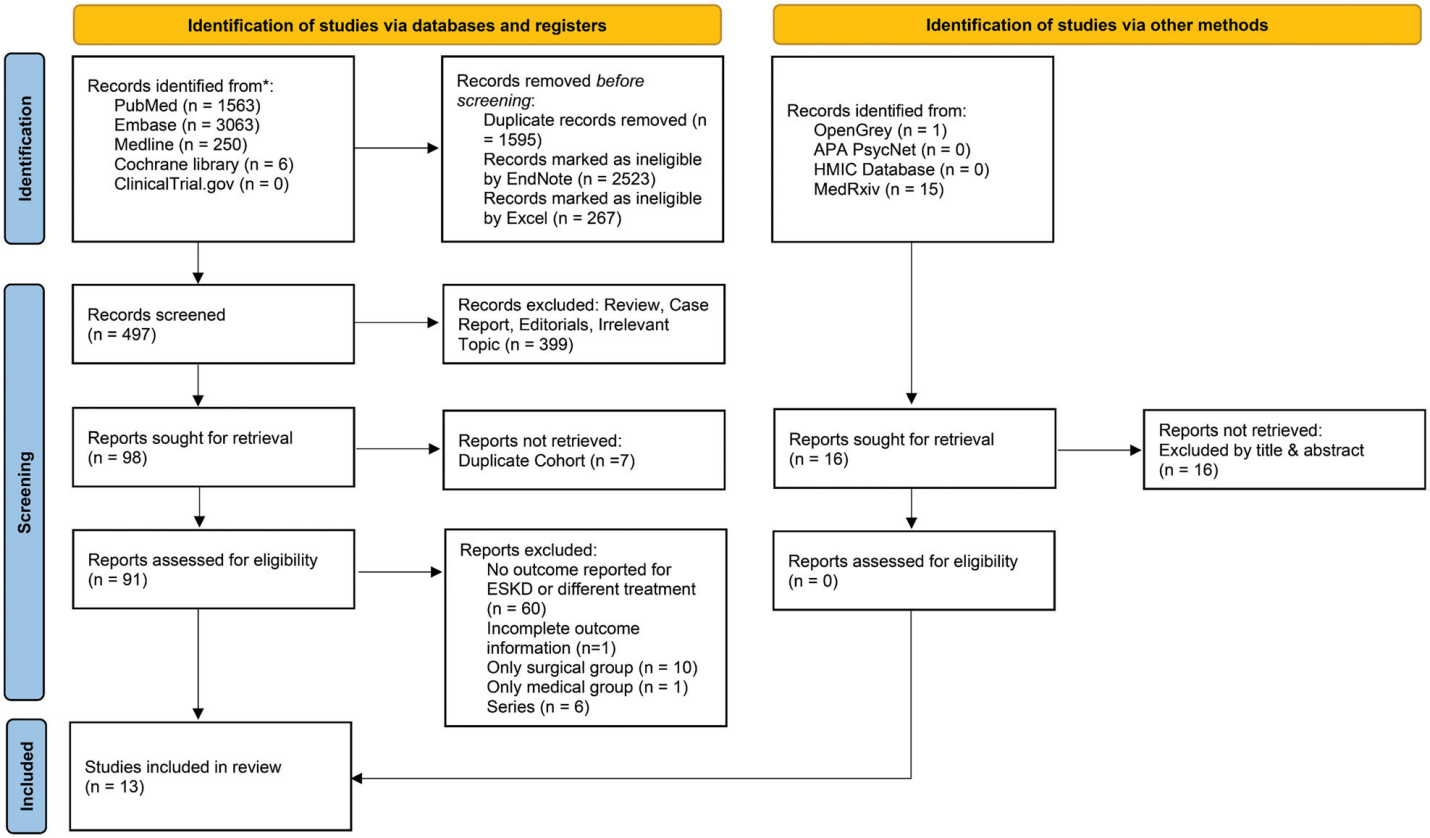
PRISMA flow diagram of literature search, exclusion, and inclusion.

Figure 1 presents the study selection process as a PRISMA 2020 flow diagram. The search identified 3281 unique articles from the online databases and registries. After the titles, abstracts, and keywords were filtered with EndNote and Excel software, 497 reports were screened. We further excluded 399 articles that were irrelevant or were reviews, editorials, or case reports and seven articles based on a duplicate cohort. In addition, 77 articles were excluded because they did not meet the eligibility criteria. No eligible articles were discovered in the gray literature search. Finally, 13 studies were included.

### Study characteristics

The included studies were published between 1997 and 2021 and comprised data from a total of 1679 patients. No randomized, controlled, or blinded trials were assessed. Only two studies were prospective cohort studies [15,16]; the remaining 11 studies employed retrospective cohort designs. Table 1 presents a summary of the study characteristics and reported outcomes [2,15–26].

**Table 1.** Characteristics of included studies.

Study	Design	Exclusion of Duke possible IE	Mean age	Male	Diabetes mellitus	Heart failure	Embolization	New-onset valve dysfunction	Prosthetic IE	Prior IE	Invasion beyond valve leaflet	Large mobile vegetation	Persistent sepsis 5–7 days despite adequate antibiotics	Follow up duration
[25]	Retrospective cohort	Not exclude	55	30%	45%	NR	40%	5%	5%	NR	NR	NR	NR	Till discharge
[19]	Retrospective cohort	Not exclude		60.70%	28.50%	42.90%	17.80%	NR	NR	NR	NR	NR	NR	1 year
[23]	Retrospective cohort	Exclude	60	52%	42%	NR	28.80%	NR	0%	12%	NR	NR	NR	5 years
[21]	Retrospective cohort	Exclude	56	45%	37.70%	30.40%	50.70%	56%	4.30%	NR	7.20%	NR	NR	Till discharge
[17]	Retrospective cohort	Exclude	57.3	47%	59%	NR	28.80%	NR	2%	10.20%	NR	NR	NR	1 years
[24]	Retrospective cohort	Not exclude	52.5	62.50%	6.20%	56.20%	18.70%	50%		NR	12.50%	37.50%	NR	Up to 21.7 months
[20]	Retrospective cohort	Exclude	55.3	52%	33.30%	NR	12%	21.40%	9.50%	9.50%	NR	NR	NR	5 years
[18]	Retrospective cohort	Not exclude	62.12	47%	59.40%	NR	NR	NR	NR	0%	NR	NR	NR	9 years
[26]	Retrospective cohort	Not exclude	NR	NR	NR	NR	29.80%	NR	NR	NR	NR	53.10%	NR	3 years
[15]	Prospective cohort	Exclude	66	69%	42.90%	40.50%	52.40%	33%	23.80%	7.10%	9.50%	NR	NR	Till discharge
[2]	Retrospective cohort	Not exclude	55	63%	43%	NR	NR	NR	NR	NR	47%	NR	NR	12 years
[22]	Retrospective cohort	Not exclude	64.2	45.70%	54.30%	31.90%	23.30%	NR	NR	NR	NR	NR	NR	5 years
[16]	Prospective cohort	Not exclude	59.9	41.40%	41.20%	25.90%	18.80%	52.50%	14%	11.80%	13.1	NR	22.40%	1 year

IE: infective endocarditis; NR: not reported.

### Associations between surgical or nonsurgical treatment and in-hospital mortality

**Figure 2.**
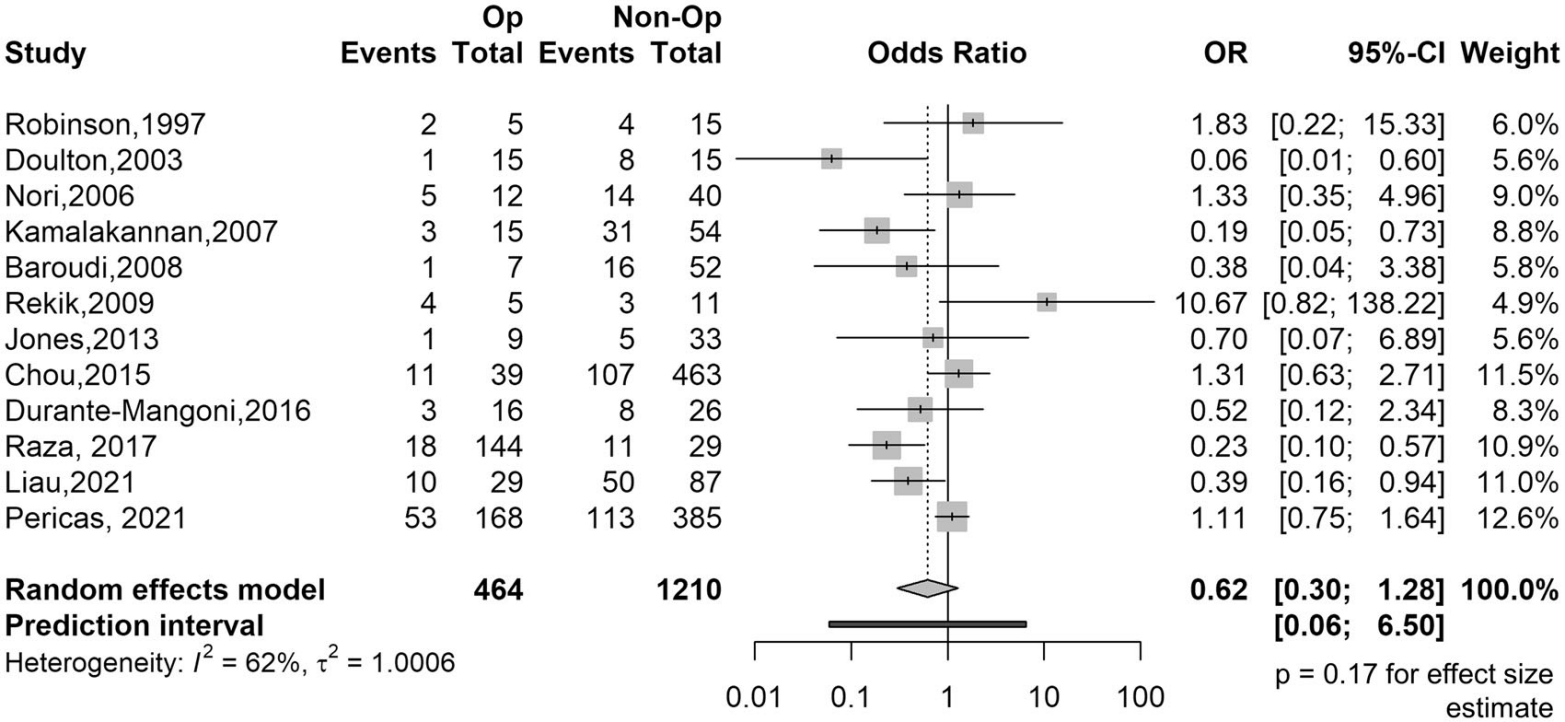
Pooled in-hospital mortality rate after infective endocarditis in patients with kidney failure.

Twelve of the 13 studies reported in-hospital mortality for the surgical and nonsurgical groups. The numbers of events and total patients in the surgically and medically treated groups are summarized in Table 2. The meta-analysis revealed that the OR for in-hospital mortality for surgical *versus* nonsurgical intervention was 0.62 (95% confidence interval [CI]: 0.30–1.28, *p* = .18, prediction interval: 0.06–6.50), with moderate heterogeneity (*I*^2^ = 62%, *p* < .01; Figure 2).

**Table 2.** Outcomes in included studies.

Author	Year	Patient numbers	In-hospital death (events/total)		30-d death (events/total)		90-d death (events/total)		180-d death (events/total)		1 year-death (events/total)	
			Surgical	Medical	Surgical	Medical	Surgical	Medical	Surgical	Medical	Surgical	Medical
Robinson	1997	20	2/5	4/15								
Doulton	2003	30	1/15	8/15	3/15	8/15						
Nori	2006	52	5/12	14/40								
Kamalakannan	2007	69	3/15	31/54								
Baroudi	2008	59	1/7	16/52	1/7	21/52					5/7	31/52
Rekik	2009	16	4/5	3/11								
Jones	2013	42	1/9	5/33	1/9	6/33	1/9	6/33			1/9	8/33
Chou	2015	502	11/39	107/463								
Powell	2015	258			11/68	61/190						
Durante-Mangoni	2016	42	3/16	8/26								
Raza	2017	173	18/144	11/29							56/126	5/18
Liau	2021	116	10/29	50/87								
Pericas	2021	553	53/168	113/385					68/168	150/385		

### Associations between surgical or nonsurgical treatment and secondary outcomes

Four studies reported 30-d mortality, and the pooled OR for surgical *versus* nonsurgical treatment was 0.36 (95% CI: 0.22–0.60, *p* = .008), with low heterogeneity (*I*^2^ = 0%, *p* < .01). No study reported 60-d mortality. Only one study reported 90-d mortality, and another one reported 180-d mortality. The OR for 90 and 180-d mortality of surgical *versus* nonsurgical intervention was 0.56 (95% CI: 0.06–5.39, *p* = .62) and 1.07 (95% CI: 0.74–1.54, *p* = .74). Three studies reported 1-year mortality, and the pooled OR of surgical *versus* nonsurgical intervention was 1.41 (95% CI: 0.20–9.93, *p* = .52), with low heterogeneity (*I*^2^ = 0%, *p* = .41; Supplemental Figure 1).

### Publication bias and risk of bias

Potential publication bias was visualized with a funnel plot (Supplementary Figure 2). Egger’s test of funnel asymmetry indicated no significant publication bias (*p* = .33; Supplementary Figure 3). We also tested funnel plot asymmetry with the trim and fill method. Using the trim and fill method did not eliminate any studies but did impute two hypothetical studies. The OR for in-hospital mortality with the trim and fill method was 0.84 (95% CI: 0.38–1.87, *p* = .65), indicating that our effect size may be slightly overestimated toward the direction of lower mortality because of the small study effect (Supplemental Figure 4). Risk of bias was assessed using the NOS because all of the included studies used nonrandomized designs. Only two studies employed adequately representative exposed cohorts; their data were derived from a nationwide or multinational database [16,18]. All the included studies adequately reported the selection of the unexposed cohort and the determination of exposure and demonstrated that outcome of interest was not present at start of study. Because the surgically and medically treated groups were not matched for age or other confounding factors, all studies got no stars in the comparability domain. All studies reported precise and valid outcomes, and all the follow-up periods were sufficient for the outcomes to occur. However, only one study achieved an adequate follow-up because of the mandatory and universal characteristics of the Taiwan National Health Insurance with nearly no loss of follow up [18]. The only large-scale prospective cohort reported 24.9% loss of follow up at 6 months following hospital discharge in the dialysis group. All other studies lack descriptions about loss of follow up (Table 3). Because all studies lack proper control for potential confounding factors, none of them get a credit in the comparability domain in NOS, and thus all included studies carried high risk of bias [16].

**Table 3.** Newcastle–Ottawa Scale risk of bias of included studies.

	Selection				Comparability	Outcome			
	Representativeness of the exposed cohort	Selection of the non-exposed cohort	Ascertainment of exposure	Demonstration that outcome of interest was not present at start of study	Comparability of cohorts on the basis of the design or analysis	Assessment of outcome	Was follow-up long enough for outcomes to occur	Adequacy of follow up of cohorts	Overall risk of bias
[25]	0	1	1	1	0	1	1	0	High
[19]	0	1	1	1	0	1	1	0	High
[23]	0	1	1	1	0	1	1	0	High
[21]	0	1	1	1	0	1	1	0	High
[17]	0	1	1	1	0	1	1	0	High
[24]	0	1	1	1	0	1	1	0	High
[20]	0	1	1	1	0	1	1	0	High
[18]	1	1	1	1	0	1	1	1	High
[26]	0	1	1	1	0	1	1	0	High
[15]	0	1	1	1	0	1	1	0	High
[2]	0	1	1	1	0	1	1	0	High
[22]	0	1	1	1	0	1	1	0	High
[16]	1	1	1	1	0	1	1	0	High

### Assessment of evidence quality and summary of findings

The primary outcome and its quality assessment were conducted by using the GRADE system. The outcomes and assessments are presented as a summary of findings in Supplementary Table 4. Owing to heterogeneous characteristics of the enrolled population and only observational studies were identified, the domains of risk of bias, inconsistency, and imprecision were judged as serious concerns. The overall certainty of evidence was considered very low.

### Subgroup and sensitivity analyses

**Figure 3.**
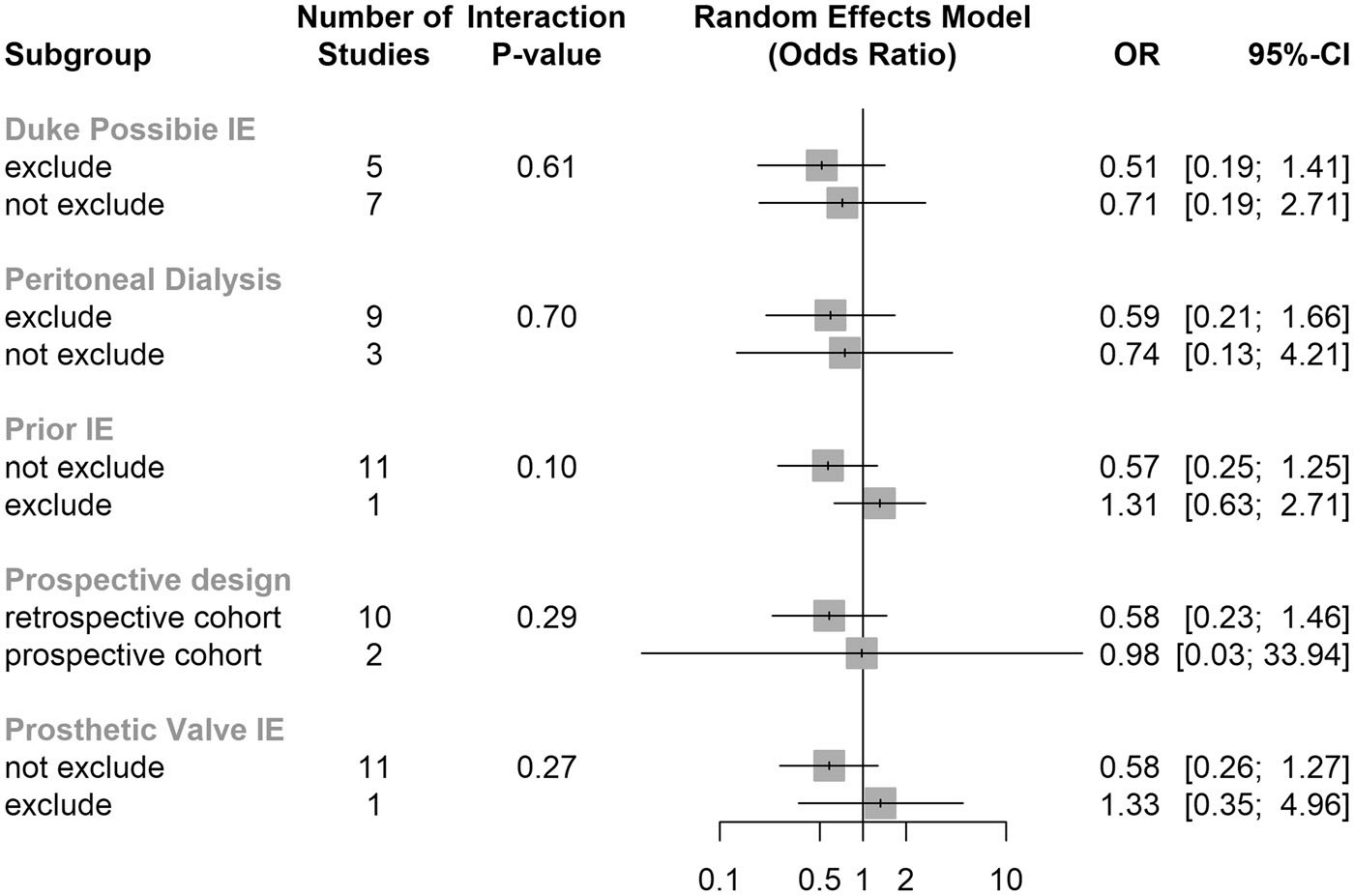
Results of subgroup analysis of the effects of potentially interacting factors on in-hospital mortality.

Subgroup analysis showed no significant differences for any subgrouping, that is, those based on (i) the exclusion of modified Duke criteria–based possible IE, (ii) the exclusion of patients undergoing peritoneal dialysis, (iii) the exclusion of patients with prior IE, (iv) the exclusion of prosthetic valve IE, and (v) study design (prospective cohort *vs.* retrospective cohort). The *p* value for interaction in above groups was all above .1 (Figure 3).

After studies with fewer than 30 patients were excluded, surgical treatment was associated with lower OR of in-hospital mortality (OR: 0.52, 95% CI: 0.27–0.99, *p* = .047, prediction interval: 0.08–3.23; Supplemental Figure 5) than was medical treatment alone, and the heterogeneity was moderate (*I*^2^ = 63%, *p* < .01). Because all of the included studies have high risk of bias, we could not conduct a sensitivity analysis by including only those studies with low or moderate risks or excluding those with a high risk. Meta-regression of in-hospital mortality on the basis of study size and year revealed no significant correlations (*p* = .49 and .94, respectively; Supplemental Figures 6 and 7).

## Discussion

Whether surgical treatment is beneficial in patients with IE undergoing dialysis remains uncertain. Here, we discovered that high-quality, randomized controlled studies are lacking, and the reports of retrospective studies may be subject to considerable bias. Our pooled results showed no statistically significant difference in in-hospital mortality between surgically and medically treated patients, with moderate heterogeneity. With small studies excluded, however, the odds of in-hospital mortality were significantly lower for surgical intervention. Although only three studies reported 30-d mortality, and the odds of this secondary outcome were also lower in the surgically treated group. These pooled results were derived from a very low certainty of evidence, as assessed by the GRADE system.

The major difficulty in comparing surgical and medical treatment in retrospective studies is the confounding factor of surgical indications; that is, patients might have undergone surgery simply because they had surgical indications, whereas those who did not receive surgery might have simply had no surgical indications. Such a potential imbalance of surgical indications makes unbiased comparison difficult. Second, the presence of multiple comorbidities could reduce the likelihood of surgical treatment. In a large study using a nationwide database in the United States, Bhatia et al. reported a lower probability of valve replacement surgery (OR: 0.82, 95% CI: 0.76–0.86) in patients with IE undergoing dialysis than in the other patients with IE; however, compared with the general population, the patients undergoing dialysis also had more comorbidities [9]. In a multinational, prospective International Collaboration on Endocarditis cohort (IE cohort), a lower likelihood of surgery for patients on dialysis was similarly observed (dialysis: 30.6% *vs.* non-dialysis: 46.2%; *p* < .001) [16]. Contraindication or high perioperative risk may also reduce the likelihood of surgical treatment. Raza et al. reported that 29 patients with IE who were on dialysis were not treated with surgery. Among these patients, 24 had surgical indications; however, 22 were too ill to receive surgery. Moreover, these 29 patients treated with antibiotics alone exhibited worse outcomes than those exhibited by patients with IE who were not on dialysis or were on dialysis but received surgical treatment [2]. The aforementioned results highlight the importance of both surgical indications and perioperative risks in a comparison of surgical *versus* nonsurgical treatment.

Another challenge is that the volume of cardiac surgery in each study hospital may be associated with different outcomes. The mortality rate after cardiac surgery in general and pediatric populations is closely linked to the surgery volume of the hospital [27–31]. This volume–outcome relationship may be linked to several factors, including the experience of the surgeons, anesthesiologists, and postoperative care team, as well as equipment maintenance [32–34]. However, during our review, hospital and surgery volume information were unavailable in all studies. Although the cardiac surgery volume of each study hospital may be retrieved indirectly from the Internet, such information would be unobtainable for nationwide databases or international registries [16,18]. Therefore, the outcomes could not be adjusted for surgery volume.

This study has several limitations. First, there are no randomized, controlled studies of the outcomes of surgical and medical treatment for IE in patients undergoing dialysis. In addition, all included studies were not primarily designed to compare the outcomes of surgical and medical intervention. Thus, outcomes were all extracted from the text, and proper matches between the two treatment arms were not performed in any of the included studies. Therefore, comparison between the two treatments was not adjusted adequately for possible confounding factors. Second, none of the included studies provided hospital or surgical volume data, which crucially affect cardiac surgery outcomes. The importance of this study, however, is its highlighting of the inadequacy of current literature on this topic and its identification of crucial information that should be included or adjusted for in future studies. In conclusion, based on a very low certainty of evidence, we found that in-hospital mortality after surgical intervention *versus* medical treatment alone do not differ for dialysis patients with IE.

## Supplementary Material

Supplemental Material

## Abbreviations

IE: infective endocarditis
PICO: population, intervention, comparison, and outcome framework
GRADE: grading of recommendations assessment, development and evaluation
NOS: Newcastle–Ottawa Scale
OR: odds ratio
CI: confidence interval
