# Supplementary material for: Surgical versus medical treatment for infective endocarditis in patients on dialysis: a systematic review and meta-analysis
Source: Ren Fail. 2022 Apr 21;44(1):706–13. doi: 10.1080/0886022X.2022.2064756 (PMC9037223; doi:10.1080/0886022X.2022.2064756)

Supplemental matarials

Supplemental Table 1. The search strategy of Pubmed

Search number	Query	Results
3	#1 AND #2	1,840
2	(infective endocarditis) OR (Endocarditis, Bacterial[MeSH Terms])	43,006
1	((((((end stage renal disease) OR (dialysis)) OR (renal failure)) OR (renal dialysis[MeSH Terms])) OR (Kidney Failure, Chronic[MeSH Terms])) OR (hemodialysis)) OR (peritoneal dialysis)	420,616

Supplemental Table 2. The search strategy and results of Embase

No.	Query	Results
#3	#1 AND #2	3028
#2	infective AND endocarditis OR (bacterial AND endocarditis)	43758
#1	end AND stage AND ('renal'/exp OR renal) AND ('disease'/exp OR disease) OR 'dialysis'/exp OR dialysis OR (('renal'/exp OR renal) AND ('failure'/exp OR failure)) OR (('renal'/exp OR renal) AND ('dialysis'/exp OR dialysis)) OR (('kidney'/exp OR kidney) AND failure, AND chronic) OR 'hemodialysis'/exp OR hemodialysis OR (peritoneal AND ('dialysis'/exp OR dialysis))	670506

Supplemental Table 3. The search strategy of Medline+Ovid

#	Query	**Results**
1	End stage renal disease.mp. or exp Kidney Failure, Chronic/	113,687
2	infective endocarditis.mp. or exp Endocarditis/	33,459
3	1 and 2	252

Supplemental Table 4

Certainty assessment							№ of patients		Effect		Certainty	Importance
**№ of studies**	**Study design**	**Risk of bias**	**Inconsistency**	**Indirectness**	**Imprecision**	**Other considerations**	**Surgical**	**Medical**	**Relative (95% CI)**	**Absolute (95% CI)**		
**Mortality**												
13	observational studies	Serious [a]	Serious [b]	not serious	serious	none	114/467 (24.4%)	371/1212 (30.6%)	**OR 0.64** (0.32 to 1.27)	**86 fewer per 1,000** (from 182 fewer to 53 more)	⊕◯◯◯ Very low	IMPORTANT
								20.0%		**62 fewer per 1,000** (from 126 fewer to 41 more)		
								60.0%		**110 fewer per 1,000** (from 276 fewer to 56 more)		

CI: confidence interval; OR: odds ratio

Explanation

a. Most studies are from retrospective cohort studies with moderate risk of bias

b. Substantial heterogeneity and low similarity of point estimates, extent of overlap of confidence intervals from enrolled studies

Supplemental figure 1. All reported outcomes in the included studies

Meta-Analysis	Number of Studies	Random Effects Model (Odds Ratio)	OR	95%-CI	p-value
In Hospital Death<br>Heterogeneity: I^2 = 62%, τ^2 = 1.0006	12		0.62	[0.30; 1.28]	0.18
30 day Death<br>Heterogeneity: I^2 = 0%, τ^2 = 0.0288	4		0.36	[0.22; 0.61]	< 0.01
90 day Death<br>Heterogeneity: I^2 = NA%, τ^2 = NA	1		0.56	[0.06; 5.39]	0.62
180 day Death<br>Heterogeneity: I^2 = NA%, τ^2 = NA	1		1.07	[0.74; 1.54]	0.74
1 year Death<br>Heterogeneity: I^2 = 0%, τ^2 = 0.3018	3		1.41	[0.20; 9.93]	0.53

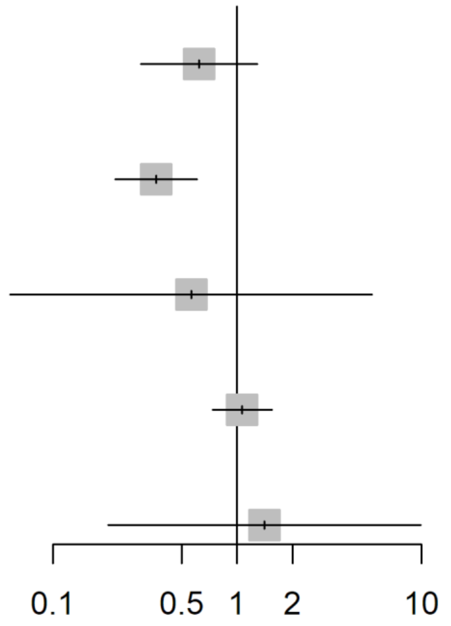

Supplemental figure 2. Funnel plot of the publication bias

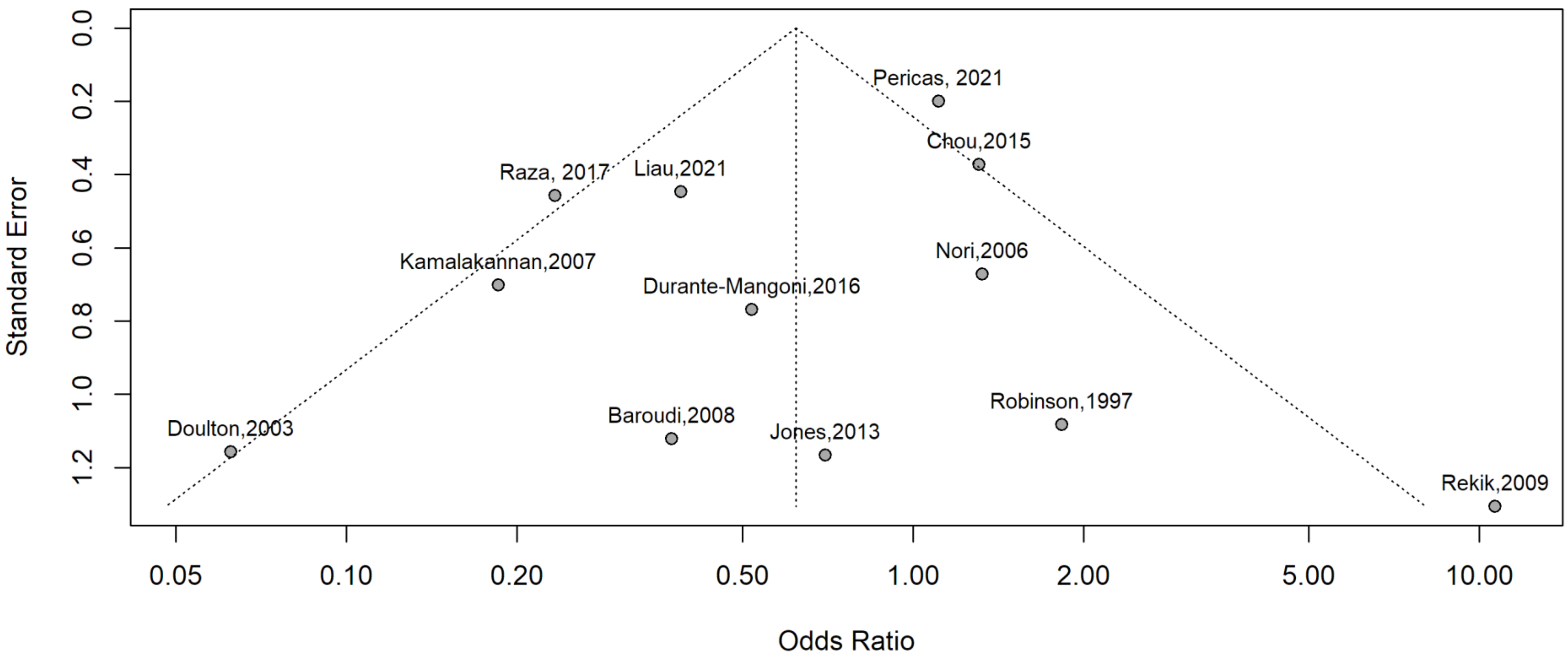

Supplemental figure 3. Plot for the Eggar test of funnel plot asymmetry

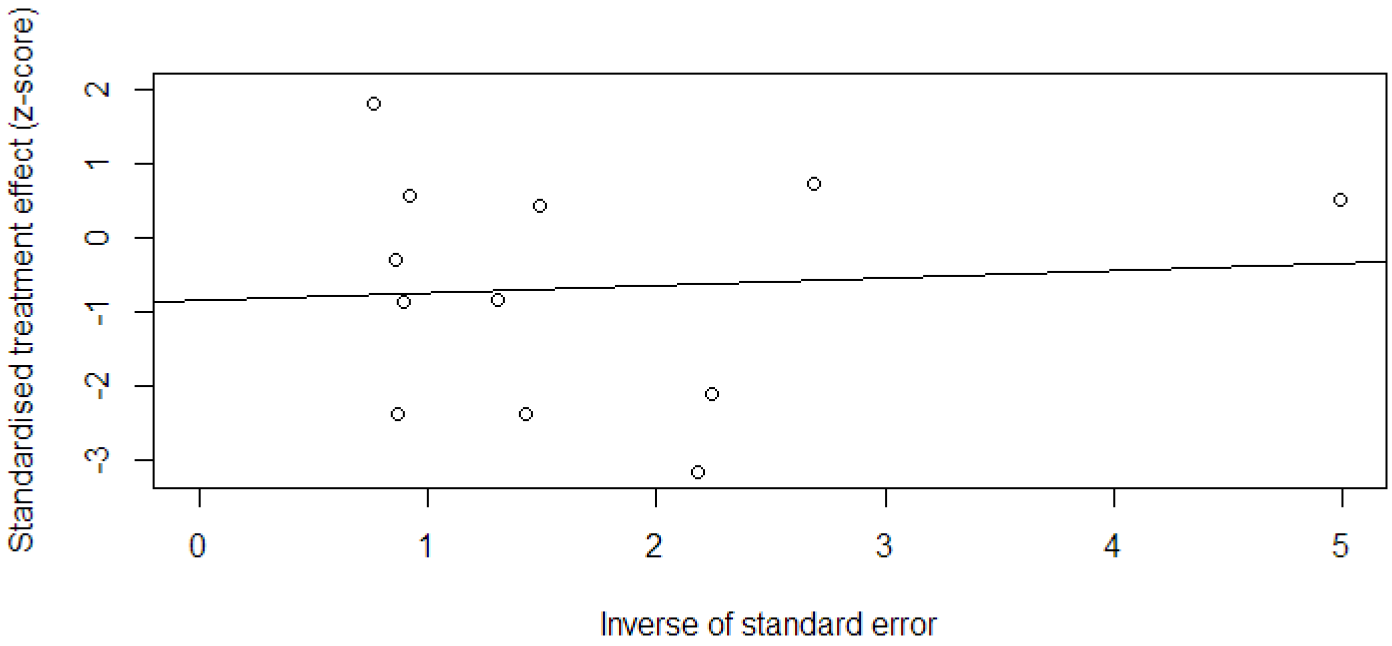

Supplemental Figure 4. Funnel plot & output of trim and fill method to assess potential publication bias

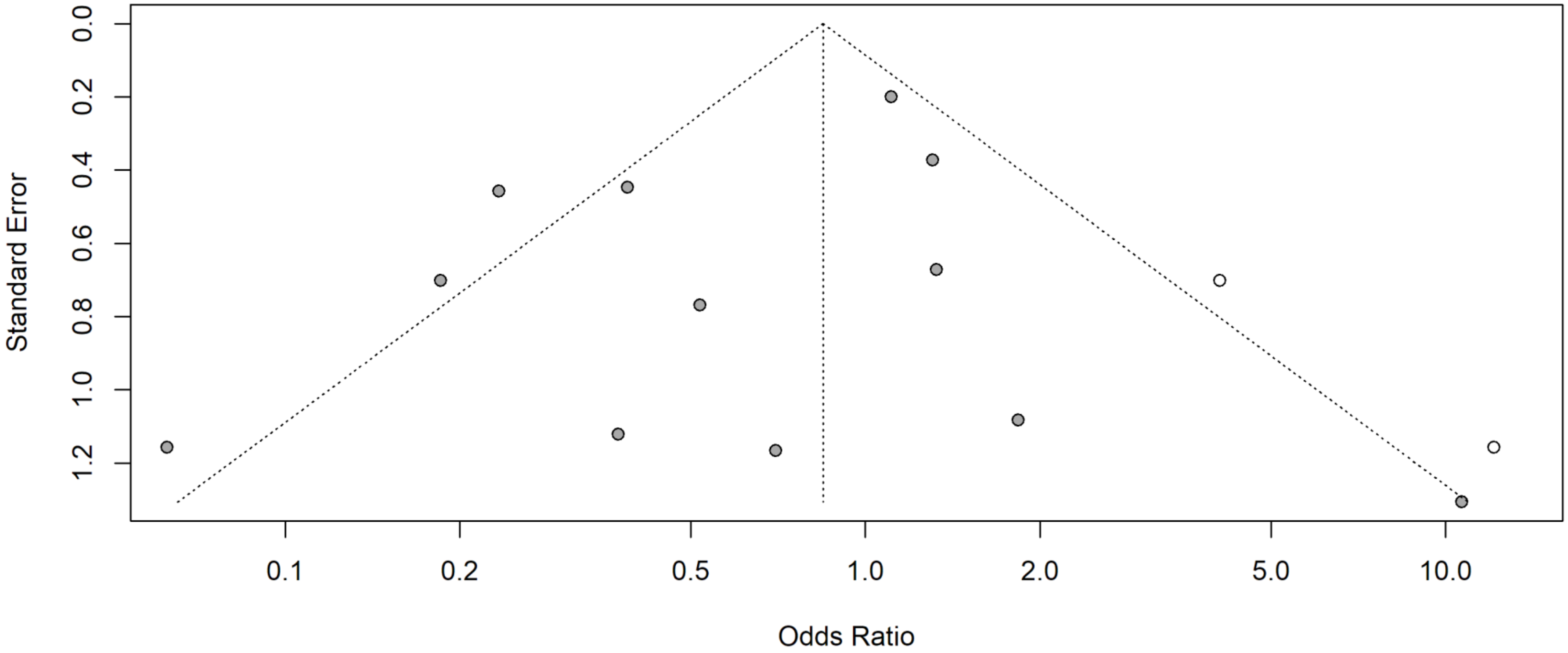

Output of the Trim and Fill Method

```
                                 OR           95%-CI %W(random)
Baroudi,2008                 0.3750 [0.0417;   3.3755]        5.5
Chou,2015                    1.3071 [0.6298;   2.7127]        9.4
Doulton,2003                 0.0625 [0.0065;   0.6039]        5.4
Durante-Mangoni,2016         0.5192 [0.1151;   2.3422]        7.3
Jones,2013                   0.7000 [0.0711;   6.8869]        5.3
Kamalakannan,2007            0.1855 [0.0469;   0.7338]        7.7
Liau,2021                    0.3895 [0.1622;   0.9350]        9.0
Nori,2006                    1.3265 [0.3548;   4.9598]        7.8
Rekik,2009                  10.6667 [0.8232; 138.2218]        4.8
Robinson,1997                1.8333 [0.2192;  15.3329]        5.7
Raza, 2017                   0.2338 [0.0952;   0.5739]        9.0
Pericas, 2021                1.1093 [0.7493;   1.6425]       10.0
Filled: Kamalakannan,2007    4.0876 [1.0332;  16.1723]        7.7
Filled: Doulton,2003        12.1311 [1.2555; 117.2122]        5.4

Number of studies combined: k = 14 (with 2 added studies)

                           OR           95%-CI     t p-value
Random effects model 0.8453 [0.3822; 1.8695] -0.46  0.6549

Quantifying heterogeneity:
 tau^2 = 1.4875 [0.2774; 4.7307]; tau = 1.2196 [0.5267; 2.1750]
 I^2 = 67.3% [42.9%; 81.3%]; H = 1.75 [1.32; 2.31]

Test of heterogeneity:
     Q d.f. p-value
 39.80   13  0.0001

Details on meta-analytical method:
- Inverse variance method
- Sidik-Jonkman estimator for tau^2
- Q-profile method for confidence interval of tau^2 and tau
- Hartung-Knapp adjustment for random effects model
- Trim-and-fill method to adjust for funnel plot asymmetry
```

Supplemental figure 5. Sensitivity analysis of the in-hospital mortality after excluding small size (n < 30) studies

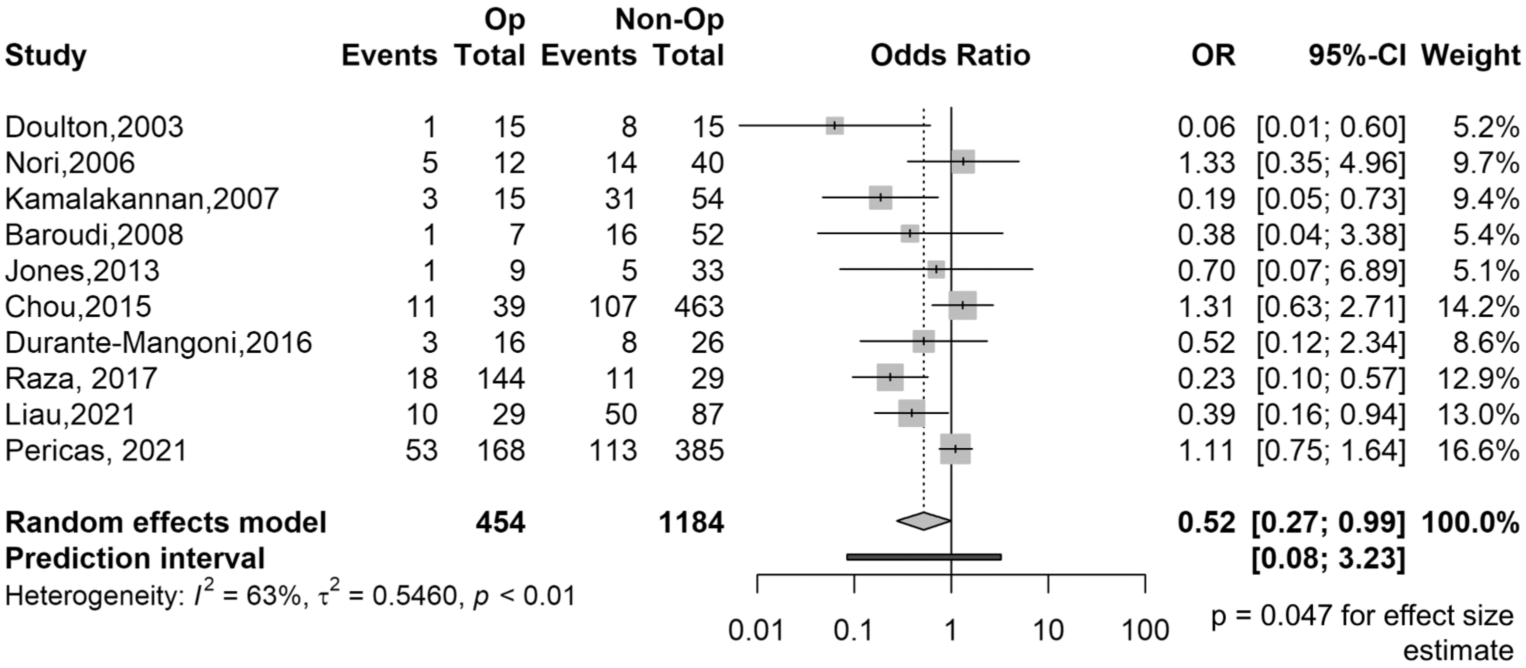

Supplemental figure 6. Meta-regression of the reported in-hospital mortality with study sample size

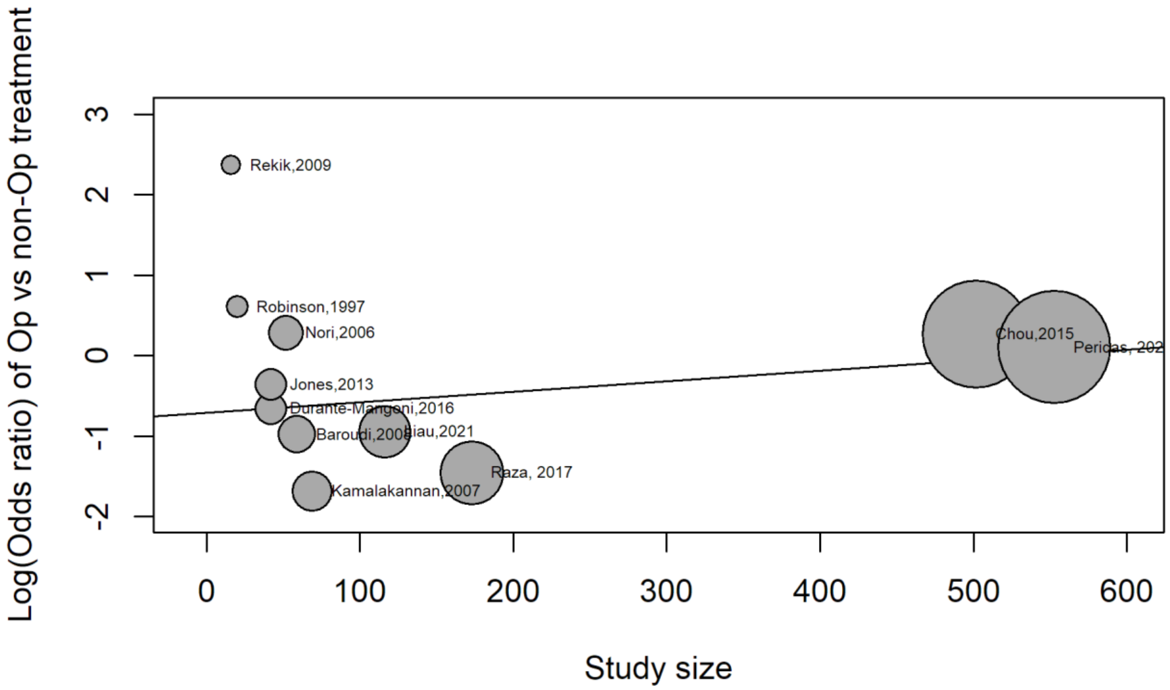

Supplemental figure 7. Meta-regression of the reported in-hospital mortality with study publication year

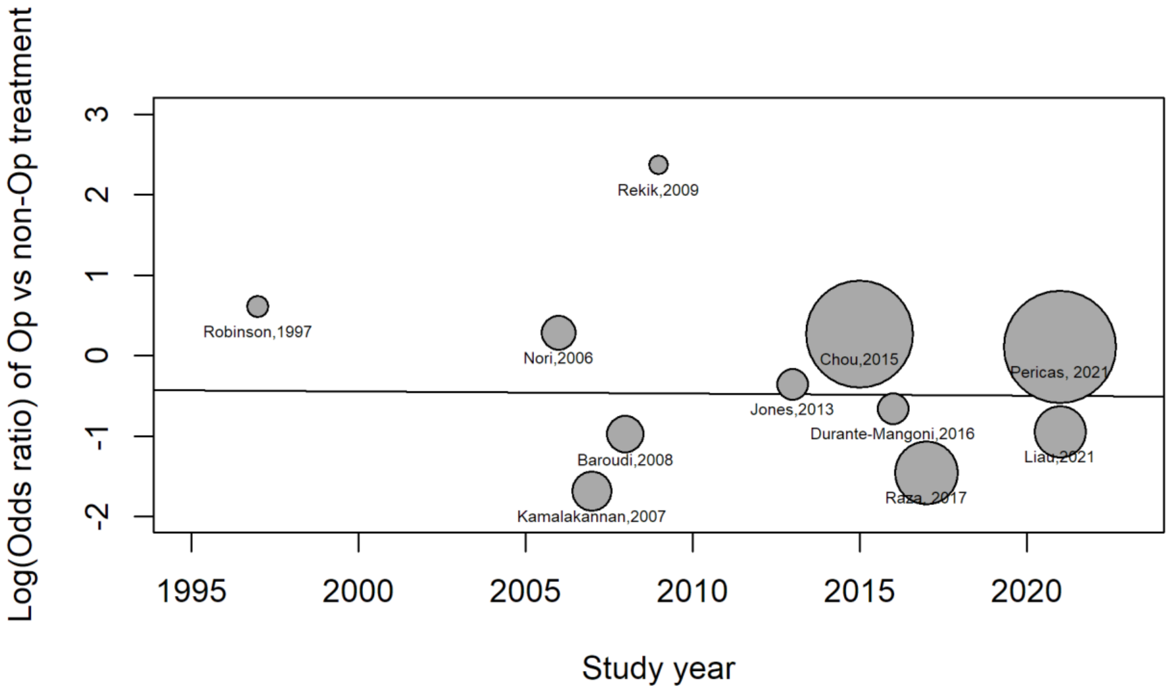

Supplement: Supplemental Material [file IRNF_A_2064756_SM4020.pdf]
